# Correction to: PhosPiR: an automated phosphoproteomic pipeline in R

**DOI:** 10.1093/bib/bbac153

**Published:** 2022-04-08

**Authors:** Ye Hong, Dani Flinkman, Tomi Suomi, Sami Pietilä, Peter James, Eleanor Coffey, Laura L Elo

**Affiliations:** Turku Bioscience Centre, University of Turku and Åbo Akademi University, Turku, Finland; Turku Bioscience Centre, University of Turku and Åbo Akademi University, Turku, Finland; Lund University, Lund, Sweden; Turku Bioscience Centre, University of Turku and Åbo Akademi University, Turku, Finland; Turku Bioscience Centre, University of Turku and Åbo Akademi University, Turku, Finland; Turku Bioscience Centre, University of Turku and Åbo Akademi University, Turku, Finland; Lund University, Lund, Sweden; Turku Bioscience Centre, University of Turku and Åbo Akademi University, Turku, Finland; Turku Bioscience Centre, University of Turku and Åbo Akademi University, Turku, Finland; Institute of Biomedicine, University of Turku, Turku, Finland

This is a correction to: Ye Hong, Dani Flinkman, Tomi Suomi, Sami Pietilä, Peter James, Eleanor Coffey, Laura L Elo, PhosPiR: an automated phosphoproteomic pipeline in R, Briefings in Bioinformatics, Volume 23, Issue 1, January 2022, bbab510, https://doi.org/10.1093/bib/bbab510.

In the originally published version of this manuscript, there was an error in the equal contribution author note. The equal contribution statement should apply to Dani Flinkman, Tomi Suomi, and Sami Pietilä only.

This error has been corrected. The publisher apologizes for the error.

