# Supplementary figures and images for: PhosPiR: an automated phosphoproteomic pipeline in R
Source: Brief Bioinform. 2021 Dec 8;23(1):bbab510. doi: 10.1093/bib/bbab510 (PMC8787428; doi:10.1093/bib/bbab510)

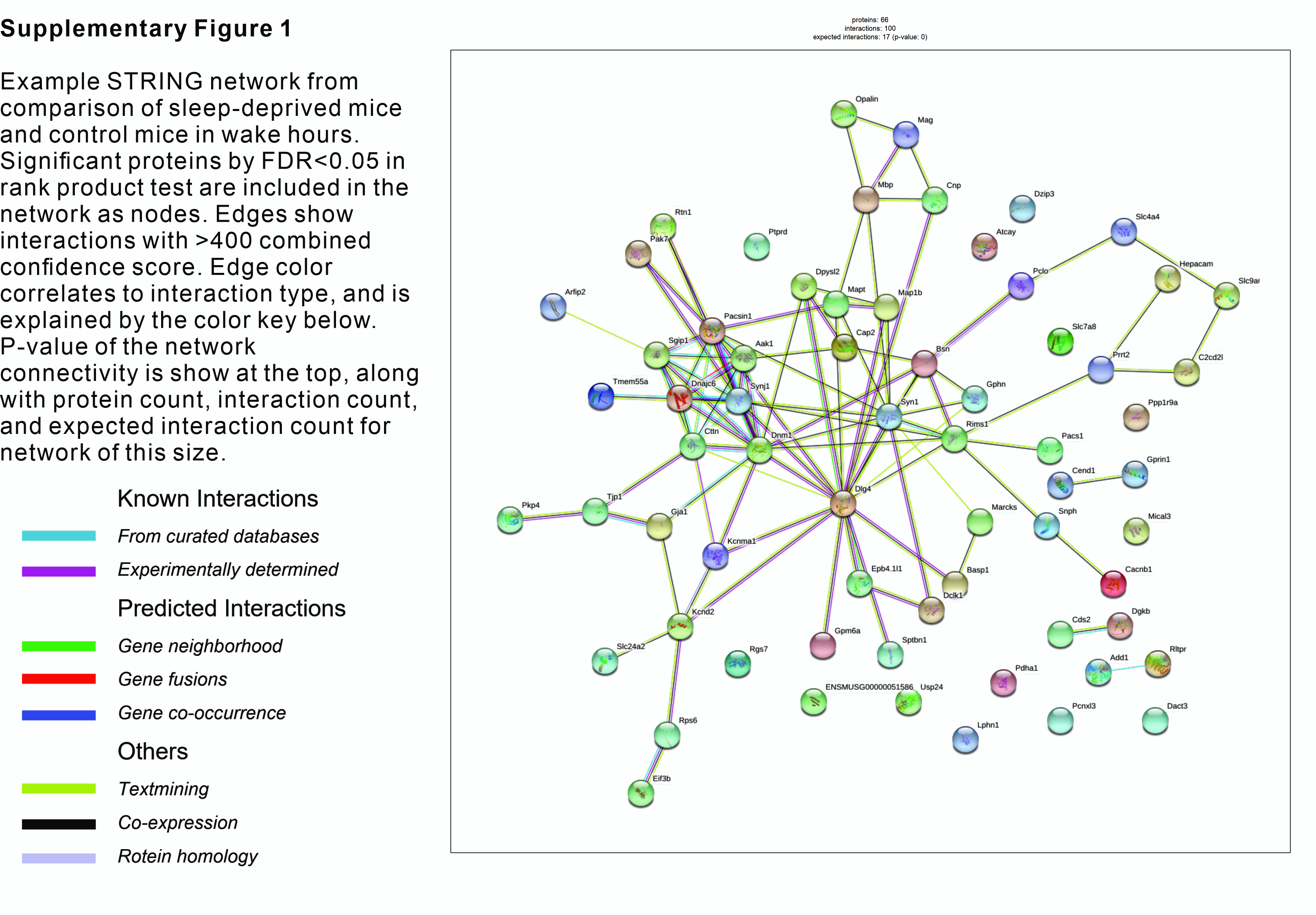

Supplement: SupplementaryFigure1_bbab510 [file supplementaryfigure1_bbab510.zip › SupplementaryFigure1_bbab510.tiff]

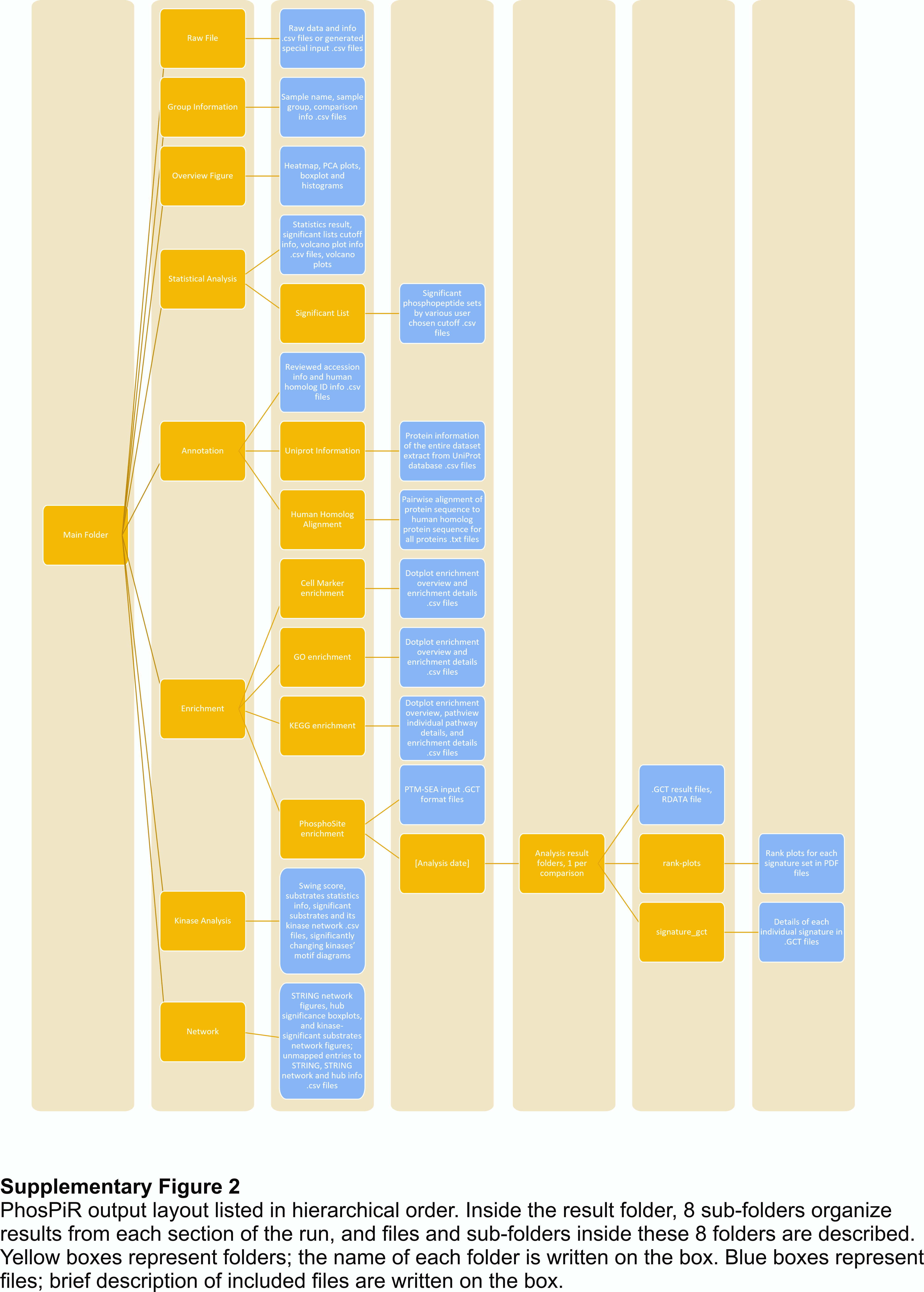

Supplement: SupplementaryFigure2_bbab510 [file supplementaryfigure2_bbab510.zip › SupplementaryFigure2_bbab510.tiff]

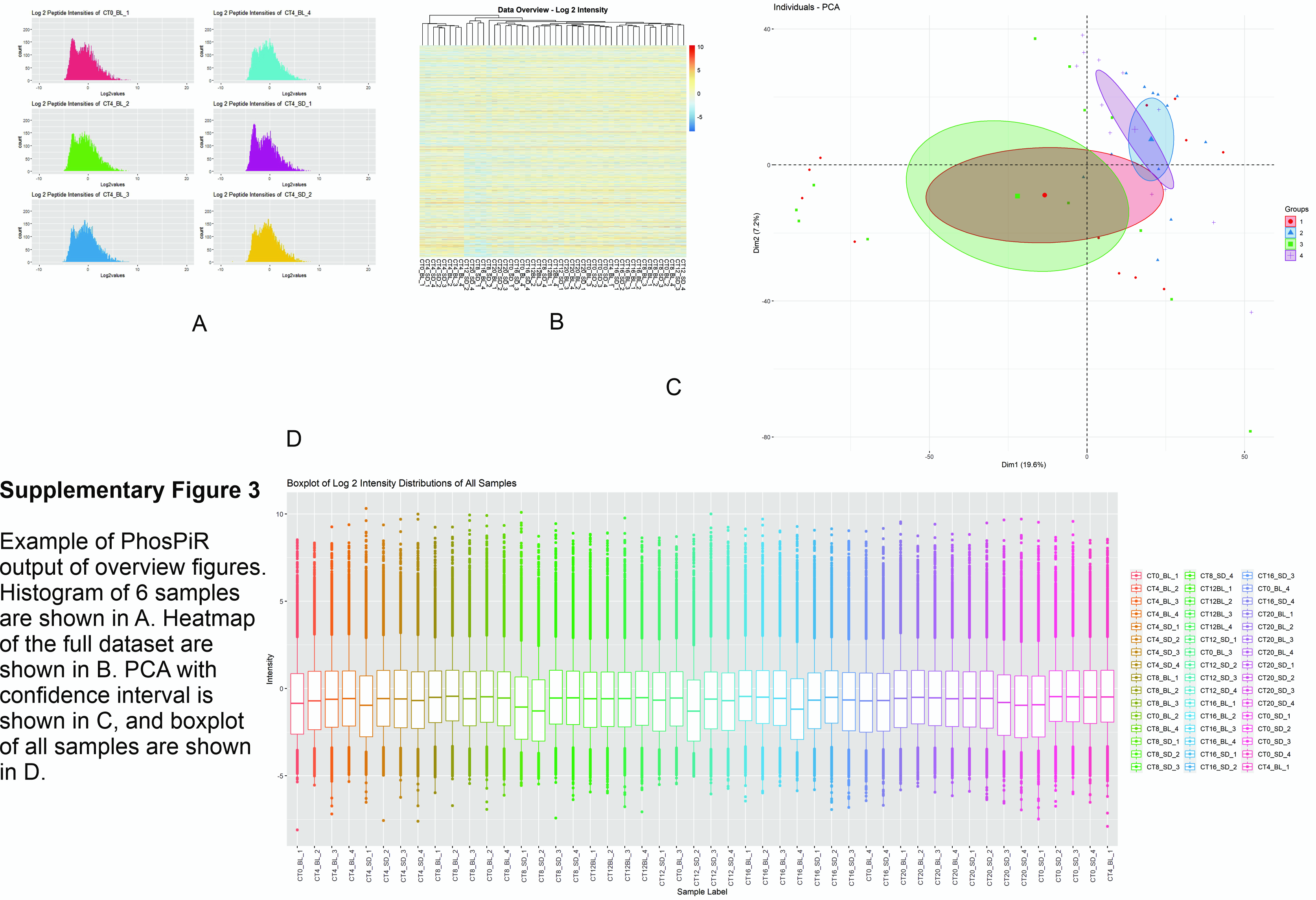

Supplement: SupplementaryFigure3_bbab510 [file supplementaryfigure3_bbab510.zip › SupplementaryFigure3_bbab510.tiff]
